# Defining neurotrauma in administrative data using the International Classification of Diseases Tenth Revision

**DOI:** 10.1186/1742-7622-8-4

**Published:** 2011-05-15

**Authors:** Amy Y Chen, Angela Colantonio

**Affiliations:** 1 Toronto Rehabilitation Institute, 550 University Ave. Toronto, Canada; 2 Department of Occupational Science and Occupational Therapy, University of Toronto.160-500 University Ave. Toronto, Canada

## Abstract

**Background:**

It is essential to use a definition that is precise and accurate for the surveillance of traumatic brain injuries (TBI) and spinal cord injuries (SCI). This paper reviews the International Classification of Diseases 10^th ^revision (ICD-10) definitions used internationally to inform the definition for neurotrauma surveillance using administrative data in Ontario, Canada.

**Methods:**

PubMed, Web of Science, Medline and the grey literature were searched for keywords "spinal cord injuries" or "brain injuries" and "international classification of diseases". All papers and reports that used an ICD-10 definition were included. To determine the ICD-10 codes for inclusion consensus across papers and additional evidence were sought to look at the correlation between the condition and brain or spinal injuries.

**Results:**

Twenty-four articles and reports were identified; 15 unique definitions for TBI and 7 for SCI were found. The definitions recommended for use in Ontario by this paper are F07.2, S02.0, S02.1, S02.3, S02.7, S02.8, S02.9, S06, S07.1, T90.2, and T90.5 for traumatic brain injuries and S14.0, S14.1, S24.0, S24.1, S34.1, S34.0, S34.3, T06.0, T06.1 and T91.3 for spinal cord injuries.

**Conclusions:**

Internationally, inconsistent definitions are used to define brain and spinal cord injuries. An abstraction study of data would be an asset in understanding the effects of inclusion and exclusion of codes in the definition. This paper offers a definition of neurotrauma for surveillance in Ontario, but the definition could be applied to other countries that have mandated administrative data collection.

## Background

The World Health Organization (WHO) has recognized the importance of surveillance systems for neurotrauma to inform prevention programs, as well as to inform the population of the significance of brain and spinal cord injuries [1]. When creating a surveillance system the definition of the condition of interest will affect the estimate and comparability of the estimate both within a country and between countries. The use of administrative medical data is attractive for neurotrauma surveillance because it is collected systematically and it is less expensive than independently collecting data. It is particularly attractive in countries with universal health insurance because data collection will also be comprehensive. However, the use of administrative data requires the translation of the clinical definition of neurotrauma to the International Classification of Diseases 10^th ^revision (ICD-10) codes, which are used in administrative data in many countries including Canada, Australia, European countries, and in the US for mortality data. It is necessary to provide information for the newest system of coding given that the ICD-10 is substantially different than ICD-9. Using ICD codes can be problematic as lack of specificity, human error, lack of time when processing medical records, and ranges of codes may affect the quality of data collection. In addition, the sensitivity of the definition depends on the codes chosen. Using a broad set of codes may incorporate more people who have suffered a traumatic brain injury (TBI) or spinal cord injury (SCI), but may also result in false positives. As a result, it is important to determine the best set of codes that will be comprehensive but also discerning of diagnosis [2]. Neurotrauma surveillance is highly important given the considerable rates of death and disability resulting from TBI and SCI worldwide, and the high costs these injuries place on health care systems and individuals alike.

Traumatic brain injury is a leading cause of death and disability among children and young adults in North America. It is estimated that 1.5 million Americans sustain a TBI annually, with an estimated 5.3 million living with permanent residual disability [3]. A study in Canada analyzed data between 1992 and 2002 and found that the age-sex standardized incidence rate of TBI hospitalizations decreased from 83.1 per 100,000 population per year to 50.4 per 100,000 population per year during this time frame, still contributing significantly to disabilities in Canada [4]. A European review of mild to severe TBI found a range of incidence from 91-105 per 100,000 population per year to a high of 546 per 100,000 population per year; the higher estimate included hospital admission, emergency department visits and death, while the lower estimate only included hospitalized patients [5].

The incidence rate of SCI in Ontario has been estimated at 24.2 per million population in 2003 and 23.1 per million population in 2006 [6]. A worldwide review of incidence studies found a range of 10.4 to 83 per million population per year [7] Although SCI does not have a large incidence rate, these injuries are typically very serious, leading to long-term disability, poor quality of life, and mortality, with high social and economic costs to the individual and the community [6]. It is estimated that in the United States, 190,000 persons live with paralysis due to a spinal cord injury, with an estimated cost of $4.5 billion annually for their care, equipment, supplies and services [8]. Although incidence is known to be significant, it is not known how accurate these numbers are and whether comparisons between the U.S. and Canada are fair due to differences in the definition used, quality of data, and methods used to collect data.

Reviews of the incidence and prevalence of neurotrauma found large ranges in estimates worldwide with broad and narrow clinical, ICD-9 and ICD-10 definitions [5,7,9]. As a result, it is important to standardize definitions and methodology to accurately estimate incidence and prevalence. In Ontario, an initiative funded by the Ontario Neurotrauma Foundation (ONF) and advised by a steering committee of stakeholders from the Ministry of Health and Long Term Care (MOHLTC), The Ontario Agency for Health Protection and Promotion (OAHPP), SMARTRISK, and the Ministry of Transportation aimed to create a neurotrauma surveillance system in order to better inform prevention in the province. The steering committee was arranged by ONF as stakeholders from the results of the surveillance system. Additional conversations were held with the Centers for Disease Control and Prevention, those involved with the Victoria Neurotrauma Initiative in Australia, as well as coding experts of the Canadian Institute for Health Information. The goals of the surveillance system are to: 1. Contribute to the reduction of injuries and related deaths in Ontario by identifying, describing and quantifying neurotrauma; 2. Collect, process and analyze summary data on neurotrauma in Ontario regarding cause of injuries, risk factors, and estimates of incidence; 3. Increase awareness of neurotrauma as a public health problem in Ontario; 4. Assist neurotrauma prevention and treatment programs by providing the data to identify trends over time, to identify high risk groups and to evaluate programs; and 5. Support neurotrauma-related research by providing data and identifying research priorities. One of the first steps towards reach these goals was to determine a definition to identify those affected by neurotrauma in mandated collection of administrative data in the emergency rooms (National Ambulatory Care Registry System, NACRS) and hospitalizations (Discharge Abstract Database).

The purposes of this study are to 1) examine the ICD-10 codes used worldwide to define traumatic brain and spinal cord injuries, 2) examine code specificity and sensitivity for both types of injury 3) use international standards to inform ICD-10 codes to use for neurotrauma surveillance in Ontario.

## Methods

### Search strategy

**Figure 1:**
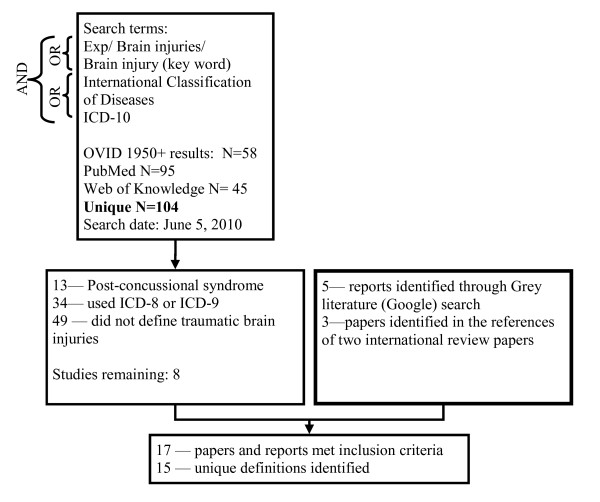
**Search terms and results for traumatic brain injury literature review of ICD-10 codes**.

This review involved searching Medline (OVID), PubMed, and Web of Knowledge using the keywords outlined in Figure 1 on June 5, 2010. In addition, references from review articles on the incidence and prevalence of TBI and SCI were examined for relevant papers.

**Figure 2:**
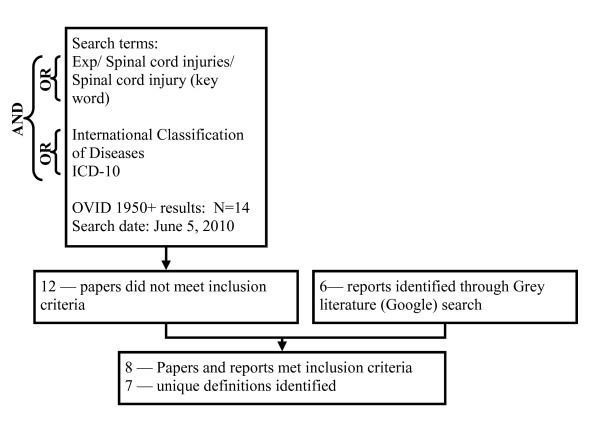
**Search terms and results for spinal cord injury literature review of ICD-10 codes**.

As incidence and prevalence rates using the ICD-10 codes are often reported in non-academic papers, a Google search was also used to identify grey literature. In total, 5 reports were found online, one from each of the following: World Health Organization (WHO), Centers for Disease Control and Prevention (CDC, U.S.), Ontario Neurotrauma Foundation (ONF, Canada), Canadian Institute for Health Information (CIHI, Canada), and National Injury Surveillance Unit (NISU, Australia) [1,3,10-12]. Four reports covered both brain injuries and spinal cord injuries; the remaining report [12] covered only brain injuries. The search terms and paper selection for traumatic brain injury codes and spinal injury codes can be found in Figures 1 and 2 respectively.

### Clinical definition of brain and spinal cord injuries

Traumatic brain injuries and spinal cord injuries have a standard definition established by the CDC and endorsed by the WHO:

"Traumatic brain injury is either:

• *An occurrence of injury to the head with at least one of the following:*

◦ *Observed or self-reported alteration of consciousness or amnesia due to head trauma*

◦ *Neurologic or neuropsychological changes or diagnoses of skull fracture or intracranial lesions that can be attributed to the head trauma*

• *Or an occurrence of death resulting from trauma with head injury or traumatic brain injury listed in the sequence of conditions that resulted in death*

Spinal Cord Injury:

• *An occurrence of an acute traumatic lesion of neural elements in the spinal canal, resulting in temporary or permanent sensory deficit, motor deficit, or autonomic dysfunction *[1]*."*

### Study selection

For "traumatic brain injuries" and for "spinal cord injuries" all papers and reports using ICD-10 to define the injury were obtained, as well as any article in which the abstract did not provide sufficient information to determine coding or population. Inclusion criteria were articles written in the English language and use of ICD-10 codes to define TBI or SCI. Codes defining the broader head injury were also included to encompass possible mild brain injury codes and to compare differences in scope. Exclusion criteria were papers using ICD-9 codes, defining post-concussive syndrome or other comorbid conditions only, and use of clinical definitions only. The information extracted from the papers and reports were 1) the codes used to define head, brain or spinal cord injuries, 2) the source of administrative data, 3) the year of the study, 4) the country where the study was conducted, 5) the study population, and 6) incidence rate or prevalence, if available.

### Defining TBI and SCI for surveillance in Ontario

Codes that have been used to define traumatic brain injuries were examined on the consistency of use across sources and countries. Codes that were consistently used and theoretically fit within the framework of brain injuries were included. Codes that were not consistently used and were not directly defining brain injuries were examined using additional evidence. Evidence was gathered on how and why codes were used in the papers/reports identified. In addition, the correlation of the condition (for example, facial fractures) to brain injuries was explored. All codes that had a strong correlation to brain injuries, fit the theoretical framework of brain injuries or were consistently used across sources were considered for inclusion in the Ontario definition by the steering committee.

For spinal cord injuries, consensus between papers and quality measures of sensitivity, specificity, positive predictive value and positive likelihood ratio informed the inclusion of codes for the definition. Ultimately the steering committee made recommendations on which codes to include beyond codes that had a consensus.

## Results

### Traumatic brain injuries

In total, 17 sources were identified that used an ICD-10 definition for traumatic brain injuries. There were two pairs of sources that used the same definition and were combined in the results table. As a result, 15 definitions can be found in Table 1, with both the ICD-10 code and the text definition of the code.

**Table 1:** Summary of ICD-10 Codes used in the literature to define TBI.

	ICD-10 Codes	WHO* [1]	CDC* [3]** Rodriguez **[29]	**Fingerhut **[18]	ABI* [12]	CIHI* [16]	**Andelic **[30]	NISU* [11]	**Crowe **[31]	**Larsson **[14]	**Deb **[13]	**Barker-Collo **[17]	**Von Wild **[32]	**Engberg **[33]	**Kleiven **[34] &**Steudel **[35]	**Peloso **[15]
Postconcussional syndrome	F07.2	-	-	-	-	X	-	-	-	-	-	-	-	X	-	-

Superficial injury of head	S00	-	-	-	-	-	-	-	-	-	X	-	-	X	-	-

Open wound(s) of scalp, open wound of eyelid and periocular area, open wound of nose, open wound of ear, open wound of cheek and temporomandibular area, open wound of lip and oral cavity	S01.0-S01.6	-	X	X	-	-	-	-	-	-	X	X	-	X	-	-

Multiple open wounds of head, open wound of other parts of head, open wound of head, part unspecified	S01.7-S01.9	X	X	X	-	-	-	-	X	-	X	X	-	X	-	-

Fracture of skull	S02.0	X	X	X	X	X	X	-	X	-	X	X	X	X	X	-
	
	S02.1	X	X	X	X	X	X	-	X	-	X	X	X	X	X	-

Fracture of nasal bones	S02.2	-	-	-	-	-	-	-	X	-	X	X	X	X	X	-

Fracture of orbital floor	S02.3	-	X	X	-	X	X	-	X	-	X	X	X	X	X	-

Facture of malar and maxillary bones	S02.4	-	-	-	-	-	-	-	X	-	X	X	X	X	X	-

Fracture of tooth	S02.5	-	-	-	-	-	-	-	X	-	X	X	X	X	X	-

Fracture of mandible	S02.6	-	-	-	-	-	-	-	X	-	X	X	X	X	X	-

Multiple fractures involving skull and facial bones	S02.7	X	X	X	X	X	X	-	X	-	X	X	X	X	X	-

Fracture of other skull and facial bones	S02.8	X	X	X	X	X	X	-	X	-	X	X	X	X	X	-

Fracture of skull and facial bones, part unspecified	S02.9	X	X	X	X	X	X	-	X	-	X	X	X	X	X	-

Dislocation, sprain and strain of joints and ligaments of head	S03	-	-	-	-	-	-	-	X S03.3	-	X	X	-	X	-	-

Injury of cranial nerves	S04	-	-	-	-	-	-	-	-	-	X	X	X	X	-	-

Injury to optic nerve and pathways	S04.0	-	X	X	-	-	-	-	-	-	X	X	X	X	-	-

Injury of eye and orbit	S05	-	-	-	-	-	-	-	-	-	X	X	-	X	-	-

Intracranial Injuries	S06	X	X	X	X	X	X	X	X	-	X	X	X	X	X	X

Concussion	S06.0	X	X	X	X	X	X	X	X	X	X	X	X	X	X	-

Diffuse brain injury, Focal brain injury	S06.2-.3	X	X	X	X	X	X	X	X	X	X	X	X	X	X	-

Crushing injury of face	S07.0	-	X	X	-	X	X	-	-	-	X	X	X	X	-	-

Crushing injury of skull	S07.1	X	X	X	-	X	X	-	-	-	X	X	X	X	-	-

Crushing injury to other parts of head	S07.8	-	X	X	-	X	X	-	-	-	X	X	X	X	-	-

Crushing injury of head, part unspecified	S07.9	X	X	X	-	X	X	-	-	-	X	X	X	X	-	-

Traumatic amputation of part of head	S08	-	-	-	-	-	-	-	-	-	X	X	-	X	-	-

Injury of blood vessels of head, not elsewhere classified	S09.0	-	-	-	-	X	-	-	X	-	X	X	X	X	-	-

Injury of muscle and tendon of head	S09.1	-	-	-	-	X	-	-	X	-	X	X	X	X	-	-

Traumatic rupture of ear drum	S09.2	-	-	-	-	X	-	-	X	-	X	X	X	X	-	-

Multiple, injuries of head	S09.7	X	X	X	X	X	X	-	X	-	X	X	X	X	-	-

Other specified injuries to the head	S09.8	X	X	-	X	X	X	-	X	-	X	X	X	X	-	-

Unspecified injuries to the head	S09.9	X	X	X	X	X	X	-	X	-	X	X	X	X	-	-

Open wounds involved head with neck	T01.0	-	X	-	-	-	-	-	-	-	-	-	-	-	-	-

Fractures involving head with neck	T02.0	-	X	-	-	X	-	-	-	-	-	-	-	-	-	-

Crushing injuries involving head with neck	T04.0	X	X	-	-	-	X	-	-	-	-	-	-	-	-	-

Crushing injuries of head with neck	T04.1-.9	-	-	-	-	-	X	-	-	-	-	-	-	-	-	-

Other injuries involving brain, cranial nerves, and spinal cord at neck level	T06.0	-	X	-	-	-	X	-	-	-	-	-	-	-	-	-

Injuries of brain and cranial nerves	T06.1-.9	-	-	-	-	-	X	-	-	-	-	-	-	-	-	-

Sequelae of open wound of head	T90.1	-	X	X	-	-	-	-	-	-	-	-	-	-	-	-

Sequelae of fracture of skull and facial bones	T90.2	-	X	X	X	-	-	-	-	-	-	-	-	-	-	-

Sequelae of cranial nerves	T90.3	-	-	-	-	-	-	-	-	-	-	-	-	-	-	-

Sequelae of fracture of eye and orbit	T90.4	-	X	X	-	-	-	-	-	-	-	-	-	-	-	-

Sequelae of intracranial injury	T90.5	-	X	X	X	-	-	-	-	-	-	-	-	-	-	-

Sequelae of other specified injuries to the head	T90.8	-	X	X	X	-	-	-	-	-	-	-	-	-	-	-

Sequelae of unspecified injuries of the head	T90.9	-	X	X	X	-	-	-	-	-	-	-	-	-	-	-

Sequelae of poisoning by drugs, medicaments and biological substances	T96	-	-	-	X	-	-	-	-	-	-	-	-	-	-	-

Sequelae of toxic effects of substances chiefly non-medicinal as to source	T97	-	-	-	X	-	-	-	-	-	-	-	-	-	-	-

Sequelae of certain early complications of trauma	T98.2	-	-	-	X	-	-	-	-	-	-	-	-	-	-	-

*Due to space constraints, not all sources are identified by author's last name, please see reference list for full citations.

The ICD-10 codes used to define TBI and head injuries were varied with the broadest definition using all head injury codes S00-S09, covering superficial injury to the head to unspecified injury to the head [13]. The broadest set of codes specific to brain injury is being used for surveillance of brain injury mortality in the U.S. as defined by the CDC [3]. S06 (intracranial injury) was the only code used universally to define traumatic brain injuries, with the exception of Larsson et al., who used only S06.2 and S06.3, and Peloso et al., who used only S06.0 [14,15].

A few codes were used by the majority of papers and reports identified. Fracture of the skull (S02.0 and S02.1) and fracture of the orbital floor (S02.3), and multiple and unspecified facial fractures (S02.7, S02.8 and S02.9) were used most consistently, in 12 out of the 17 papers, with the exception of S02.3, which was used in 10 papers. Crushing injuries of the face, skull and head (S07.0, S07.1, S07.8, and S07.9) were also used in at least 7 of the papers. Lastly multiple, other and unspecified injuries to the head (S09.7, S09.8, and S09.9) were used in at least 8 of the papers. Sequelae codes (T codes) were used in four of the papers/reports in the U.S., Canada and Denmark.

### Spinal cord injuries

In total, 8 sources were identified that met the inclusion criteria and defined SCI using ICD-10 codes. A pair of sources used the same definition and as a result 7 definitions were identified and can be found in Table 2.

**Table 2:** Summary of ICD-10 Codes used in the literature to define SCI.

	ICD-10 Code	**WHO **[1]	**Jaglal **[6]**RHSCIR **[27]	**Fingerhut **[18]	**Smartrisk-Compass **[36]	**CIHI **[16]	**NISU-Australia **[11]	**Hagen **[28]
Spastic tetraplegia	G82.4	-	-	-	-	-	-	X

Fractures to cervical spine	S12.0-12.7	-	-	-	-	X	-	X (12.0, 12.2)

Fracture of neck, part unspecified	S12.9	-	-	-	-	X	-	-

Traumatic rupture of cervical intervertebral disk	S13.0	-	-	-	-	-	-	X

Dislocation of other and unspecified parts of neck	S13.2	-	-	-	-	-	-	X

Sprain and strain of cervical spine	S13.4	-	-	-	-	-	-	X

Injury of cervical spinal cord	S14.0	X	X	X	X	X	X	X
	
	S14.1	X	X	X	X	X	X (14.10-14.13)	X

Injury of nerve root of cervical spine	S14.2*	X	-	-	-	-	-	-

*Functional level of cervical spinal cord injury* from ICD-10-AM (Australia only)	S14.70-S14.78	-	-	-	-	-	X	-

Traumatic amputation at neck level	S18	X	-	-	-	-	-	-

Multiple injuries of neck	S19.7*	X	-	-	-	-	-	-

Fracture of thoracic vertebra, multiple fractures of thoracic spine, fracture of sternum	S22.0-22.1	-	-	-	-	X	-	X (S22.0)

Dislocation of thoracic vertebra	S23.1	-	-	-	-	-	-	X

Injury of thoracic spinal cord	S24.0	X	X	X	X	X	X	X
	
	S24.1	X	X	X	X	X	X (24.10-24.12)	X

Injury of nerve root of thoracic spine	S24.2*	X	-	-	-	-	-	-

*Functional level of thoracic spinal cord injury* from ICD-10-AM (Australia only)	S24.70-S24.77	-	-	-	-	-	X	-

Fracture of lumbar vertebra, sacrum or coccyx	S32.0-32.2	-	-	-	-	X	-	-

Dislocation of lumbar vertebra	S33.1	-	-	-	-	-	-	X

Injury of lumbar spinal cord	S34.0	X	X	X	X	X	X	X
	
	S34.1	X	X	X	X	X	X	X

Injury of nerve root of lumbar and sacral spine	S34.2*	X	-	-	-	-	-	-

Injury of cauda equine	S34.3	X	X	X	-	-	-	X

Functional level of lumbar spinal cord injury	S34.7	-	-	-	-	-	X	-

Crushing injuries involving head with neck, or thorax, abdomen, lower back or pelvis	T04.0-T04.1*	X	-	-	-	-	-	-

Traumatic amputations involving other combinations of body regions (e.g. abdomen or thorax	T05.8*	X	-	-	-	-	-	-

Injuries of brain and cranial nerves with injuries of nerves and spinal cord at neck level	T06.0-	X	X	-	X	-	X	X

Injury of nerves and spinal cord involving other multiple body regions	T06.1	X	X	-	X	-	X	X

Fracture of spine, level unspecified	T08	-	-	-	-	X	-	-

Injury of spinal cord, level unspecified	T09.3	X	-	X	-	-	X	X

Injury of unspecified nerve, spinal nerve root, and plexus of trunk	T09.4*	X	-	-	-	-	-	-

Traumatic amputation of trunk, level unspecified	T09.6	X	-	-	-	-	-	-

Sequelae of fracture of spine	T91.1	-	-	-	-	-	-	X

Sequelae of injury of spinal cord	T91.3	-	-	X	-	-	X	X

*These codes were identified to be less specific to spinal cord injury and require additional abstraction from medical records by the WHO.

Codes used to define spinal cord injuries were more consistent across sources. Injury to the cervical spinal cord (S14.0, S14.1), injury to the thoracic spinal cord (S24.0, S24.1), and injury to the lumbar spinal cord (S34.0, S34.1) were universally used to define spinal cord injury. Injury to the cauda equine (S34.3) was used by 5 of the 8 papers.

### Incidence rates from identified sources

Identified papers were from Europe, North America and Australia. Data sources from each paper/report were comprehensive hospital registers, emergency room records and death records. Not all papers included prevalence and incidence rates, with each country varying by their method of administrative data collection. The lowest rates of brain/head injury incidence reported were age-specific rates reported to be 40.1 per 100,000 in those aged 20-39 in Canada (head injury hospitalizations); the highest rate of incidence was reported at 349.2 per 100,000 in New Zealand (brain injury hospitalizations) [16,17]. However, incidence rates are not comparable across countries as different definitions and different age groups are reported. A summary of sources reporting incidence or prevalence rates of traumatic brain injury and head injury are summarized in table 3. Incidence rates for SCI were not reported universally. The number of cases identified in the relevant sources can be found in table 4.

**Table 3:** A Summary of Sources Identified on Traumatic Brain Injury Codes.

Author (Year)	Country	Data Source	Type of Data	Study Population	Incidence/Prevalence
Barker-Collo (2009) [17]	New Zealand	National Health Information Service, 1997-2004	Hospital discharge data	--	Crude incidence rate per 100,000 in 2002/2003: 349.2, age-standardized incidence rate: 342 (337-349)

von Wild (2008) [32]	Germany	Hospitals from two regions, 2000-2001	Patient data	n = 7,010; all ages	Incidence per 100,000 for the study period: 332

Fingerhut (2006) [18], Minino (2006) [37]	U.S.	NCHS, 2002	Multiple cause of death file	n = 247,195	Prevalence of TBI among all injury deaths in 2002: 26.9%

Rodriguez (2006) [29]	U.S.	NCHS and Oklahoma Injury Surveillance System database	Mortality or hospital discharge data	n = 1,656	-

Deb (1999) [13]	U.K.	Accident & emergency department case register	Hospital admission data	n = 410,500	688 incident cases

Larsson (2010) [14]	Sweden	Social insurance and hospital data, 1999-2002	hospital admission data	-- 16-64 years of age	250 incidence cases

Peloso (2004) [15]	Sweden	Hospital Discharge Register at the National Board of Health and Welfare, 1987-2000	Hospital discharge data		209 per 100,000 (men), 148 per 100,000 (women)

Crowe (2009) [31]	Australia	Chart review, Royal Children's Hospital, Melbourne, 2004	Specific hospital data	ED visits total = 54,233	1,115 incident cases in children

CIHI (2007) [16]	Canada	NACRS, 2003-2004	Hospital discharge data	--	16,811 incident cases; Age specific rates per 100,000: 62.5 (0-19) 40.1 (20-39) 35.6 (40-59) 90.1 (60+)

**Table 4:** A Summary of Sources Identified on Spinal Cord Injury Codes.

Author (Year)	Country	Data Source	Type of Data	Study Population	Incidence/Prevalence
Jaglal (2009) [27]	Canada	NACRS, DAD, 2003-2006	emergency room and hospital discharge data	--	559 cases

Fingerhut (2006) [18]	U.S.	NCHS, 2002	Multiple cause of death file	n = 247,195	Prevalence of SCI among all injury deaths in 2002: 0.7%

CIHI (2007) [16]	Canada	NACRS, 2003-2004	Hospital discharge data	**--**	10400 incident cases of spinal injuries

Hagen (2008) [28]	Norway	Electronic database of hospital records	Hospital records (discharge data)	3 counties, n = approx 1 million	--

NISU (2009) [11]	Australia	Victoria hospitalizations, 2004-05	Hospital separations (discharge data)	--	Incidence: 1756 cases

Compass (2007) [36]	Canada	DAD, 2004-05	Hospital discharge data	--	Incidence: 296 cases

NACRS, National Ambulatory Care Reporting System; DAD, Discharge Abstract Database; NCHS, National Center for Health Statistics

### Traumatic brain injury code evidence

Overall there was a consensus on the use of S06 intracranial injuries in the definition of brain injuries. Codes in the S00, S03, S04, S08 chapters were used by papers/reports encompassing head injuries. Codes S01, S02, S07 and S09 were used by many of the papers/report (see table 1), as a result, the merit of each code was explored. The summary of what sources were used and the results can be found in table 5.

**Table 5:** Investigation of codes to be included for the definition of traumatic brain injuries for surveillance in Ontario.

Block of Code	Consensus	Sources of Evidence	Evidence
S00 - Superficial injury to head	Only used to define head injury	--	--

S01 - Open wound to head	Recommended by CDC, WHO, Fingerhut, used by those defining head injury	Evidence from Fingerhut paper and discussion with CDC.	Used by coroners to code those with gunshot wound to head [18].

S02 - Facial Fractures	At least one S02 code included by 12 out of 16 papers.	Papers on correlation between specific facial fractures and brain injuries.	Retrospective data from a trauma database found that in blunt trauma, more people had TBI among those with facial fracture compared to those without TBI (p < 0.001) [22] Those with facial fractures have a higher incidence of severe head injury and score lower on the Functional Independence Measure (FIM) on discharge compared to those with no facial fracture [22] The odds ratio of a brain injury was 24.4 for an orbital fracture, and 135 for a maxillary fracture compared to those without a brain injury [20] 70.2% of those with an orbital facial fracture and 75.5% of those with a zyoma fracture sustained a brain injury in a study of those hospitalized from a motorcycle accident [21]

S03-S05, S08 -- Dislocation, sprain and strain of joints and ligaments of head; injury to cranial nerves; injury to eye and orbit; Traumatic amputation of part of head	Only used to define head injury	--	--

S07 - Crush injuries to the head	9 out of 17 papers used at least one S07 code	Papers on correlation between crush injuries and brain injuries. Only case studies were found.	A case study of eight children who suffered crush head injuries all suffered from identifiable cerebral trauma [23]. Another case study of seven children found that they all experiencing multiple fractures of the head, including facial, orbits, frontal, sphenoid, ethmoid, occipital, and temporal bones [38].

S09 - multiple and unspecified injuries to the head	11 out of 17 papers used at least one S09 code	Data from ICD-9 studies on the inclusion of unspecified codes and data from ABI dataset on the specificity and sensitivity of codes used.	The ABI Dataset found that there is at least a 3 fold increase in numbers of cases by adding S09 codes [12]. The CDC found that used 959.01 in ICD-9 the use of this code has grown steadily and may lead to lower specificity of the code (positive predictive value of 20%) [26]

### Spinal cord injury code evidence

For spinal cord injuries there was international consensus among the papers/reports identified for the inclusion of codes S14.0, S14.1, S24.0, S24.1, S34.0, and S34.1. One of the papers identified evaluated the codes based on sensitivity and specificity in administrative data in Norway. The method used was to cast the net broadly encompassing all codes that may indicate spinal cord injury. A reabstraction of records with those codes using medical records was performed either confirming or rejecting with the diagnosis of a spinal cord injury. Sensitivity, specificity, positive predictive value and positive likelihood ratio were calculated for each individual code and also two sets of codes. The values for each are presented in table 6.

**Table 6:** Sensitivity, Specificity, Positive Predictive Value and Positive Likelihood Ratios calculated by Hagen in Norway [26]

	ICD-10 Code	Sensitivity	Specificity	Positive Predictive Value	Positive Likelihood Ratio
Spastic tetraplegia	G82.4	0.143	0.965	0.500	4.124

Injury of cervical spinal cord	S14.0	0.152	0.988	0.762	13.196
	
	S14.1	0.229	1.000	1.000	n/a

Injury of thoracic spinal cord	S24.0	0.105	0.998	0.917	45.362
	
	S24.1	0.152	0.998	0.941	65.981

Injury of lumbar spinal cord	S34.0	0.009	0.995	0.333	2.062
	
	S34.1	0.067	1.000	1.000	n/a

Injury of cauda equine	S34.3	0.057	0.998	0.857	24.743

Sequelae of injury of spinal cord	T91.3	0.333	0.991	0.897	36.083

Combination 1: G82.4, S14.0-1, S24.0-1, S34.0-1, S34.3, T91.3	See below	0.867	0.935	0.765	13.402

Combination 2: S14.0-S14.1, S24.0- S24.1, S34.1, S34.3, T91.3	See below	0.838	0.972	0.880	30.241

From these results, it is evident that out of the codes identified by Hagen the best combination uses the codes S14.0-S14.1, S24.0-S24.1, S34.1, S34.3, and T91. These codes have a sensitivity of 0.838, meaning using all these codes would capture 83.8% of all spinal cord injuries identified in Norway. This also means that just over 16% of those with spinal cord injuries would not be captured. These codes have a specificity of 0.972%, meaning 97.2% of injuries identified are actually spinal cord injuries. Finally, the codes have a positive predictive value of 0.880, meaning that of those who are identified as having a spinal cord injury, 88% actually has a spinal cord injury.

## Discussion

To our knowledge, this is the first paper that systematically reviews ICD-10 codes worldwide for the purpose of neurotrauma surveillance. In light of the wider range of codes used, we make recommendations for surveillance in Ontario that may be applicable to other jurisdictions utilizing mandatory, linkable administrative data for surveillance.

Despite a recommended definition by the WHO, there is no international consensus on the ICD-10 codes to use for the definition of traumatic brain injuries. In designing a neurotrauma surveillance system in Ontario, the definition used would have important implications for data usage and interpretation, as a broad definition may result in inflated numbers and a narrow definition may exclude some who may have experienced a brain injury. A balance of sensitivity and positive predictive value is needed for a reasonable estimate of true incidence. The diverse definitions used worldwide may also reflect the range and quality of data (e.g. the way data are reported and used).

To decide which codes should be included for an Ontario neurotrauma surveillance system, we undertook a comparison of codes used internationally and gathered evidence on the reasoning for the inclusion of codes, and on the correlation between codes and brain injury (See Tables 5 and 6).

### TBI codes

For brain injuries, the ICD-10 intracranial codes of S06 are widely accepted international codes for inclusion in brain injury surveillance. For the code S01, inclusion in the CDC definition was based on mortality data in which the majority of cases were suicide deaths by firearms [18]. In the Canadian Coding Standards (2009), S01, open wounds code "animal bites, cuts, lacerations, avulsion of skin and subcutaneous tissue and puncture wounds with or without penetrating foreign body....do not include...[those that] involve deeper tissue." [18]. As a result, brain injuries do not fit under these coding standards; therefore comparison with US data are not applicable [18,19]. The steering committee decided not to include S01 in Ontario surveillance data because most of the data collected are not mortality data.

The strong correlation between specific facial fractures and brain injury led to the recommendation by the steering committee to include all S02 codes involving skull fractures (S02.0, S02.1, S02.7, S02.8, and S02.9) for the definition of brain injuries. In addition, the S02 code for fracture of the orbital floor (S02.3) is included in the definition used in Ontario [20-22]. The inclusion of the code S02.3 was based on three separate studies: first a case-control study of those injured in a bicycle accident found that those with brain injuries had 24.4 times the odds of also having an orbital fracture compared to those without a brain injury [20]; a study of those hospitalized for a motorcycle collision found that 70.2% of those with a orbital facial fracture also sustained a brain injury [21]; finally, a retrospective data study using a trauma database found that more people had TBI among those with facial fracture compared to those without facial fracture (p < 0.0010) [22]. (See table 5 for more information). The steering committee felt that there was insufficient evidence to support the inclusion of the following facial fractures: S02.2 (fracture of the nasal bones), S02.4 (fracture of malar/maxillary bones) S02.5 (fracture of the tooth); S02.6 (fracture of the mandible). More research is warranted to justify the inclusion of these codes.

Injury to the optic nerve and pathways (S04) was also utilized in 6 of the 16 papers. This code does conceptually fit with intracranial injury; however, with the advice of the steering committee it was excluded due to differences in treatment and outcomes in comparison to what are clinically considered brain injuries. The exclusion of this code will be discussed further with stakeholders in Ontario.

Crush injuries are defined as static forces of >200 ms applied over a wide area. This static force deforms the cranium which may result in damage to intracranial structures [23,24]. The Canadian coding standards dictates that crush injuries should be identified as comorbid conditions or main/other problem with significant injuries [19]. The correlation between crush injuries to the head and brain injury led to the inclusion of crush injuries to the skull, S07.1 codes (crushing injury to the skull), to be included in the definition of TBI for the surveillance of brain injury in Ontario. Crush injuries to the face and head are less specific in description and no literature was identified to support its inclusion in neurotrauma.

Multiple and unspecified head injuries are coded as S09.7-S09.9. The experience of members of the investigative team in Ontario was that there would be a large increase in numbers of cases by adding this code [12]. In addition, although the CDC has recommended that this code be included in TBI surveillance (959.01 in ICD-9), over time the increased use of this code has resulted in misclassification [25]. In fact, including the unspecified code 959.01 in CDC surveillance led to an increase use of the code for brain and non-brain injury patients, resulting in lower specificity of the code for TBI (positive predictive value of 20%) [26]. The exclusion of S09.9 means that cases of TBI will not be counted and the overall rate derived from this method would be a conservative estimate. Up to 20% of all S09.9 codes may be mild TBIs that would missed with the exclusion of S09 in a TBI definition based on a previous report [26]. More work, however, needs to be done in the Canadian context to reveal the number and nature of TBIs coded using the unspecified, S09 codes and to better understand how many injuries would be excluded if this code is not used.

Sequelae codes were included as this would allow any patients that may not have been captured in their first visit to the hospital to be captured in the future. Only sequelae codes that specifically related to the skull and facial bones were included in the definition of brain injuries for surveillance in Ontario (T90.2 - sequelae of fracture of skull and facial bones and T90.5 - sequelae of intracranial injury, and F02.7 - post concussive syndrome). In Ontario, all hospitals are mandated to collect data from the emergency room (NACRS) and upon discharge (DAD). These two databases, NACRS and DAD, can be merged by a scrambled ID of patients based on their health insurance card number allowing them to be tracked through their care. As a result, those who visit the emergency room or are admitted due to a previous brain injury can be tracked over time and counted only once for incidence. The inclusion of sequelae codes are not recommended for countries where data cannot be tracked by person as this may result in double-counting.

Overall, the codes used to define brain injuries in the Ontario surveillance system were F07.2, S02.0, S02.1, S02.3, S02.7, S02.8, S02.9, S06 (.0-.9), S07.1, T90.2, or T90.5.

It is important to note that our definition of TBI tended to be conservative for case ascertainment, which is most relevant for program planning of post-injury care.

### SCI codes

The literature review found higher consistency of ICD-10 codes used to define SCI in the sources identified. A combination of codes was used to define spinal cord injuries for surveillance in Ontario based on the quality of data study by Hagen (2008) and the codes used by Jaglal (2009) [6,27]. From Hagen's study, codes S14.0, S14.1, S24.0, S24.1, S34.1, S34.3, and T91.3 were used in the definition of spinal cord injury for surveillance in Ontario [28]. The code S34.0 was used universally by the sources identified and aligns with Canadian databases of the Rick Hansen spinal cord database and research by Susan Jaglal. As a result, this code was also included in the Ontario definition [27]. The sequelae codes T06.0 and T06.1, injuries of the brain and cranial nerves with spinal cord injury at neck, and injury of nerves and spinal cord involving other multiple body regions were included because of their relevance and to align with Canadian databases [6,27].

Overall, the codes used to define spinal cord injuries in the Ontario surveillance system were S14.0, S14.1, S24.0, S24.1, S34.1, S34.3, and T91.3.

## Conclusions

This review makes a recommendation for a standard Canadian definition of neurotrauma based on experience and studies of injury correlations. Since the international literature is inconsistent in defining brain and spinal cord injuries using ICD-10 codes, the definition used for neurotrauma (TBI and SCI) surveillance in Ontario was chosen based on international consensus between studies/reports, a review of the correlation between codes and brain injuries, a review of the reason for inclusion of codes in definitions used in other countries, and ultimately a discussion with the steering committee. Reviews of incidence and prevalence of TBI and SCI recognize the importance of an international consensus on definitions of these injuries based on evidence to allow comparability of data across countries.

Future research should explore the quality of data in Canada using a reabstraction study similar to the one conducted by Hagen in Norway [28]. Estimates of sensitivity, specificity, positive predictive value and likelihood ratio will quantify how incidence rates would be affected by the inclusion or exclusion of specific codes. Replication of coding quality studies in other countries would inform definitions to be used internationally. This study provides a preliminary definition based on codes used internationally and available quality indicators and known correlations. Moving forward, there are certain codes that still require discussion on the merit of inclusion. In particular the inclusion of unspecified codes is contentious due to inflated rates. From the data gathered by the CDC and previous reports, the proposed definition in Ontario will result in an underestimate in the rate of brain injuries by not including specific S09 codes in the definition. However, the decision not to include S09 codes will likely increase the specificity of the definition. From our experience and from recommendations from the CDC and journal articles, we decided not to include the codes in S09 in our definition. A study on the specificity and sensitivity of codes, in particular S09 would be helpful in making a more accurate estimate of rate. If the majority of any of the S09 codes, with a particular interest in S09.9, are in fact brain injuries from abstraction of medical records in accordance to clinical definitions, the inclusion of the S09 code or an adjustment in our rate estimates would be warranted. Our current definition is a conservative definition that would be applicable in other countries that similarly use ICD-10 administrative codes for morbidity and mortality. Continued research, discussion and monitoring of trends over time are recommended for both changes in coding practices and injury patterns. Ongoing review of coding practices, injury patterns and evolving evidence of related injuries should be conducted to review these recommendations over time.

## Competing interests

The authors declare that they have no competing interests.

## Authors' contributions

AYC conducted the review and compiled the content of each of the papers. AYC wrote and edited the manuscript. AC was responsible for the overall conceptualization of the project and secured funding for the project. She managed the review and checked the results of the literature review. AC also contributed to the methods of the search and made significant contributions to the discussion and conclusion sections of the manuscript. All authors edited and reviewed the final manuscript.
